# Can quantitative analysis of multi-parametric MRI independently predict failure of focal salvage HIFU therapy in men with radio-recurrent prostate cancer?

**DOI:** 10.1016/j.urolonc.2021.04.017

**Published:** 2021-12

**Authors:** Arnas Rakauskas, Taimur T. Shah, Max Peters, Jagpal S. Randeva, Feargus Hosking-Jervis, Michael J. Schmainda, Clement Orczyck, Mark Emberton, Manit Arya, Caroline Moore, Hashim U. Ahmed

**Affiliations:** a Imperial Prostate, Division of Surgery, Department of Surgery and Cancer, Imperial College, London, UK; b Department of Radiotherapy, University Medical Center Utrecht, Heidelberglaan, Utrecht, The Netherlands; c Imaging Biometrics, LLC, Wisconsin, USA; d Department of Urology, University College London Hospitals NHS Foundation Trust, London, UK

**Keywords:** HIFU, Prostate cancer, Radiotherapy, Quantitative parameters, Salvage treatment

## Abstract

**Objectives:**

Focal salvage HIFU is a feasible therapeutic option in some men who have recurrence after primary radiotherapy for prostate cancer. We aimed to determine if multi-parametric quantitative parameters, in addition to clinical factors, might have a role in independently predicting focal salvage HIFU outcomes.

**Methods:**

A retrospective registry analysis included 150 consecutive men who underwent focal salvage HIFU (Sonablate500) (2006-2015); 89 had mpMRI available. Metastatic disease was excluded by nodal assessment on pelvic MRI, a radioisotope bone-scan and/or choline or FDG PET/CT scan. All men had mpMRI and either transperineal template prostate mapping biopsy or targeted and systematic TRUS-biopsy. mpMRI included T2‐weighted, diffusion‐weighted and dynamic contrast‐enhancement. Pre-HIFU quantitative mpMRI data was obtained using Horos DICOM Viewer v3.3.5 for general MRI parameters and IB DCE v2.0 plug-in. Progression-free survival (PFS) was defined by biochemical failure and/or positive localized or distant imaging results and/or positive biopsy and/or systemic therapy and/or metastases/prostate cancer‐specific death. Potential predictors of PFS were analyzed by univariable and multivariable Cox-regression.

**Results:**

Median age at focal salvage HIFU was 71 years (interquartile range [IQR] 65–74.5) and median PSA pre-focal salvage treatment was 5.8ng/ml (3.8-8). Median follow-up was 35 months (23-47) and median time to failure was 15 months (7.8–24.3). D-Amico low, intermediate and high-risk disease was present in 1% (1/89), 40% (36/89) and 43% (38/89) prior to focal salvage HIFU (16% missing data). 56% (50/89) failed by the composite outcome. A total of 22 factors were evaluated on univariable and 8 factors on multivariable analysis. The following quantitative parameters were included: Ktrans, Kep, Ve, Vp, IS, rTTP and TTP. On univariable analysis, PSA, prostate volume at time of radiotherapy failure and Ve (median) value were predictors for failure. Ve represents extracellular fraction of the whole tissue volume. On multivariable analysis, only Ve (median) value remained as an independent predictor.

**Conclusions:**

One pharmacokinetic quantitative parameter based on DCE sequences seems to independently predict failure following focal salvage HIFU for radio-recurrent prostate cancer. This likely relates to the tumor microenvironment producing heat-sinks which counter the heating effect of HIFU. Further validation in larger datasets and evaluating mechanisms to reduce heat-sinks are required.

## Highlight

• Quantitative mpMRI parameters predict failure of salvage HIFU in radiorecurrent prostate cancer
• Tumour microenvironment might produce heat-sinks which counter the effect of HIFU
• Ve value measured in the DCE sequence of the mpMRI is an independent predictor of treatment failure

## 1 Introduction

Currently, most men with recurrence after radiotherapy are managed with watchful waiting and androgen deprivation therapy (ADT), a palliative treatment strategy which carries significant side effects [1]. Focal salvage HIFU is used in some centers as it may offer disease control in men at high risk of progression whilst minimizing complications compared to salvage prostatectomy [2,3] although recent publications suggest that with a robotic approach the complications are lower than previously reported [4,5].

Focal salvage therapy aims to treat the area of recurrent disease rather than the entire prostate gland. A recent publication on outcomes of salvage HIFU after external beam radiotherapy (EBRT) failure have shown low risk of urinary incontinence, erectile dysfunction and rectal injury [2]. However, about a half of patients can have biochemical failure after focal salvage treatment.

Multi-parametric (mpMRI) is an important tool in detecting local recurrence after EBRT [6]. The hypervascular nature of malignancy means dynamic contrast-enhanced (DCE) sequences provide better contrast enhancement and accurate delineation of the tumor despite fibrosis [7]. A meta-analysis has shown mpMRI with DCE is better at diagnosing recurrence after EBRT with sensitivity and specificity of 90% and 81% respectively compared with 82% and 74% for T2WI alone [8]. Apparent diffusion coefficient (ADC) sequences might also provide important information. Quantitative analysis of diffusion within a tumor show significantly lower ADC values in malignant prostate tissue compared to healthy tissue in untreated patients [9].

A recent analysis of mp‐MRI, with the quantitative maps resulted in optimal distinction between tumor and benign voxels in patients after ERBT [10]. These parameters might also help to describe the tumor micro-environment and predict possible effects of heat-sink during HIFU, which might be detrimental to its success. Our aim was to assess the role of quantitative mpMRI parameters of mpMRI, in addition to clinical factors, in predicting outcomes after focal salvage HIFU.

## 2 Materials and methods

### 2.1 Study population

This is a retrospective analysis of 150 patients who underwent focal salvage HIFU procedures between 2006 and 2015. The inclusion and exclusion criteria to the study are listed in Table 1.

**Table 1:** Inclusion and exclusion criteria

Inclusion criteria	Exclusion criteria
Diagnosis of radiorecurrent PCa	Missing clinical data
Primary treatment was EBRT	No mpMRI scan or missing ADC map, DCE or DWI
Underwent focal salvage HIFU after EBRT failure	mpMRI >2 years old
Pre-HIFU mpMRI scan on PACS	Inadequate scan due to poor contrast enhancement
	The HIFU treated radio-recurrent PCa tumor Is not demonstrable on mpMRI or obscured by artefact
	Missing data regarding site of failure
	< 12 month follow-up

From this cohort patients were selected who met our inclusion criteria with a diagnosis of radiorecurrent PCa where primary treatment was EBRT and subsequent focal salvage HIFU. The aim of this study was to determine if quantitative mpMRI parameters, performed prior to salvage HIFU, might predict the outcome in addition to clinical parameters.

Patients who had cytoreduction with ADT pre-HIFU had PSA levels recorded prior to cytoreduction as their actual pre-HIFU levels were artificially low. In all instances, the ADT treatment was used for a short period of time between the confirmation of the recurrence (after MRI) and the HIFU treatment. ADT treatment was discontinued on the day of surgery in all cases. Those patients on ADT for six or more months had it stopped and the PSA monitored until it rose back to the pre-ADT levels before imaging and re-biopsy them again. Patients receiving ADT treatment were not excluded from the analysis and were followed with identical protocol as the patients who were not receiving ADT prior to HIFU.

The choice between a focal and hemi-ablation was decided by the surgeon according to the tumor extent and location. Broadly speaking, small tumors in the peripheral zone were treated with a more focally ablative approach whilst larger tumors extending into the anterior part of the prostate were treated with hemi-ablation. Biochemical failure was defined using the Phoenix criteria as either no nadir level achieved post-HIFU or PSA nadir +2 ng/ml. Imaging and histological failure was defined as in-field recurrence (tumor recurrence at HIFU treatment site) and metastatic disease on any imaging modality or biopsy. Repeat mpMRI, bone scan or Choline PET CT were the imaging modalities used in the follow-up if progression was suspected. Any out-of-field recurrence (recurrence with new tumor focus outside HIFU treatment area in the prostate gland) was not classified as failure for our analysis as treatment of the targeted lesion was deemed to be successful. PCa is known to be a multifocal disease in up to 60-90% of cases so out-of-field failures could not be attributed to the HIFU treatment of the original and/or index lesion with certainty [11]. Development of metastases was defined as focal HIFU failure even if further evidence of local recurrence was not available. Although micrometastases may have been present at the time of salvage treatment, assessment of this was not within the scope of this study. Death within the study timeframe was counted as failure. Time to failure was calculated from time of HIFU date to the type of failure detected first. Length of follow-up was from HIFU operation date to last clinic date or last PSA result.

### 2.2 mpMRI analysis

Images were initially reviewed on a PACS viewer (Picture archiving and communications system) before being uploaded to Horos DICOM Viewer software for processing (Horos version 3.3). Only the PCa tumors treated with HIFU were of interest. These were contoured to calculate the quantitative imaging data. The following parameters were obtained:

Tumor volume: Due to the loss of zonal anatomy secondary to previous EBRT, tumors were contoured on the DCE where they were clearly visible and well demarcated from surrounding prostate tissue. A ROI (region of interest) was contoured on each slice of the DCE and tumor volume calculated by the software (Fig.s 1 & 2).

**Fig. 1:**
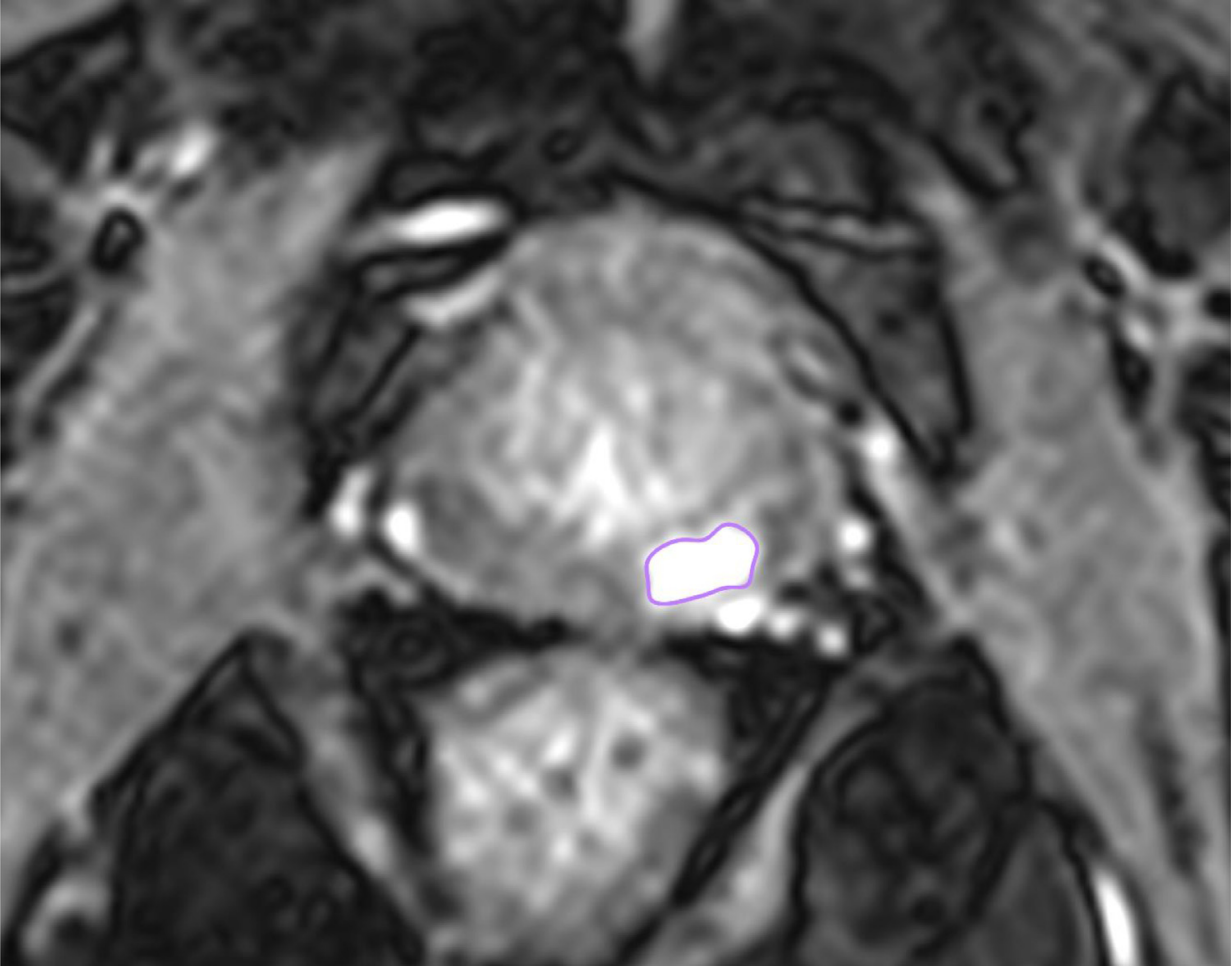
contouring the tumor in Axial DCE sequence, a high contrast uptake is present at the 4-6 o'clock position in the peripheral zone identifying tumor contoured in purple.

**Fig. 2:**
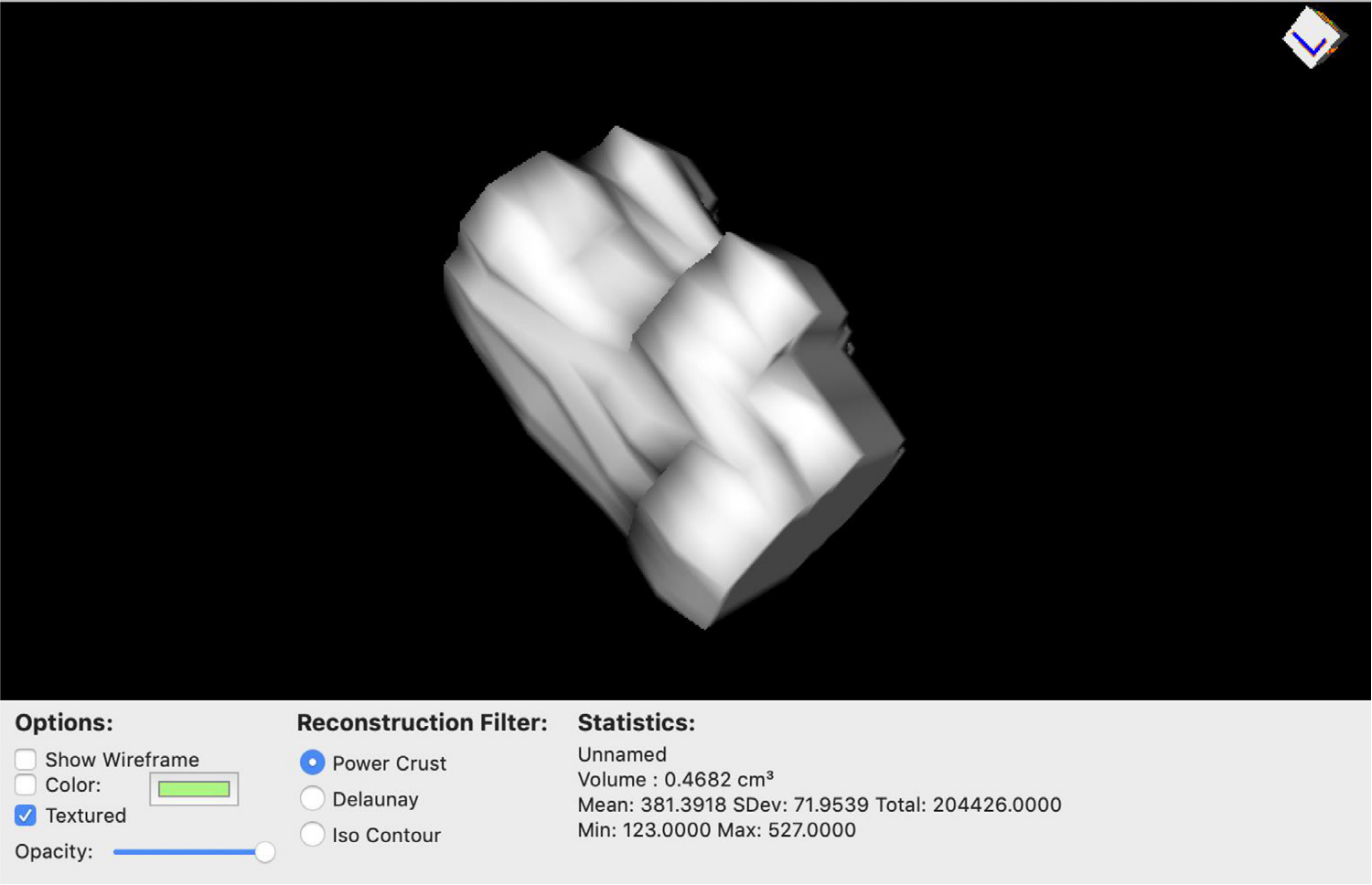
ROI tumor volume calculation with 3D rendering of tumor in Horos.

ADC value: this was calculated by contouring the ROI on the MRI ADC map and DCE side-by-side on as many slices where the HIFU treated lesions were visible. The software automatically generated the mean and standard deviation of the ADC value.

### 2.3 Quantitative mpMRI parameters

Quantitative pharmacokinetic modelling of DCE using the Tofts model, first described in 1989, is a popular method [12]. It is a two-compartment analysis where calculations are made as contrast rapidly diffuses from the vascular compartment to the tissue compartment (interstitial space) during ‘wash-in’ and returns into the vascular compartment during ‘wash-out’. Signals calculated on serial images can be plotted on a signal vs. time curve by measuring the quantity of contrast in the tissue compartment based on the fraction of space occupied by tumor, tumor volume and concentration of contrast in the tissue compartment. Three key parameters exist in empirical quantitative analysis of DCE in relation to the signal vs. time curve; interstitial space volume (Ve), volume transfer constant (Ktrans) and rate constant (kep). Ktrans and kep are both measured as contrast per unit volume of tissue per minute. Ve establishes the peak signal on the curve and tumors with greater Ve take longer for contrast to reach peak volumes in the interstitial space. Kep measures the rate constant as contrast is reabsorbed from interstitial space into the vascular compartment which is equal to Ktrans/Ve and determines the overall curve shape.

**Fig. 3:**
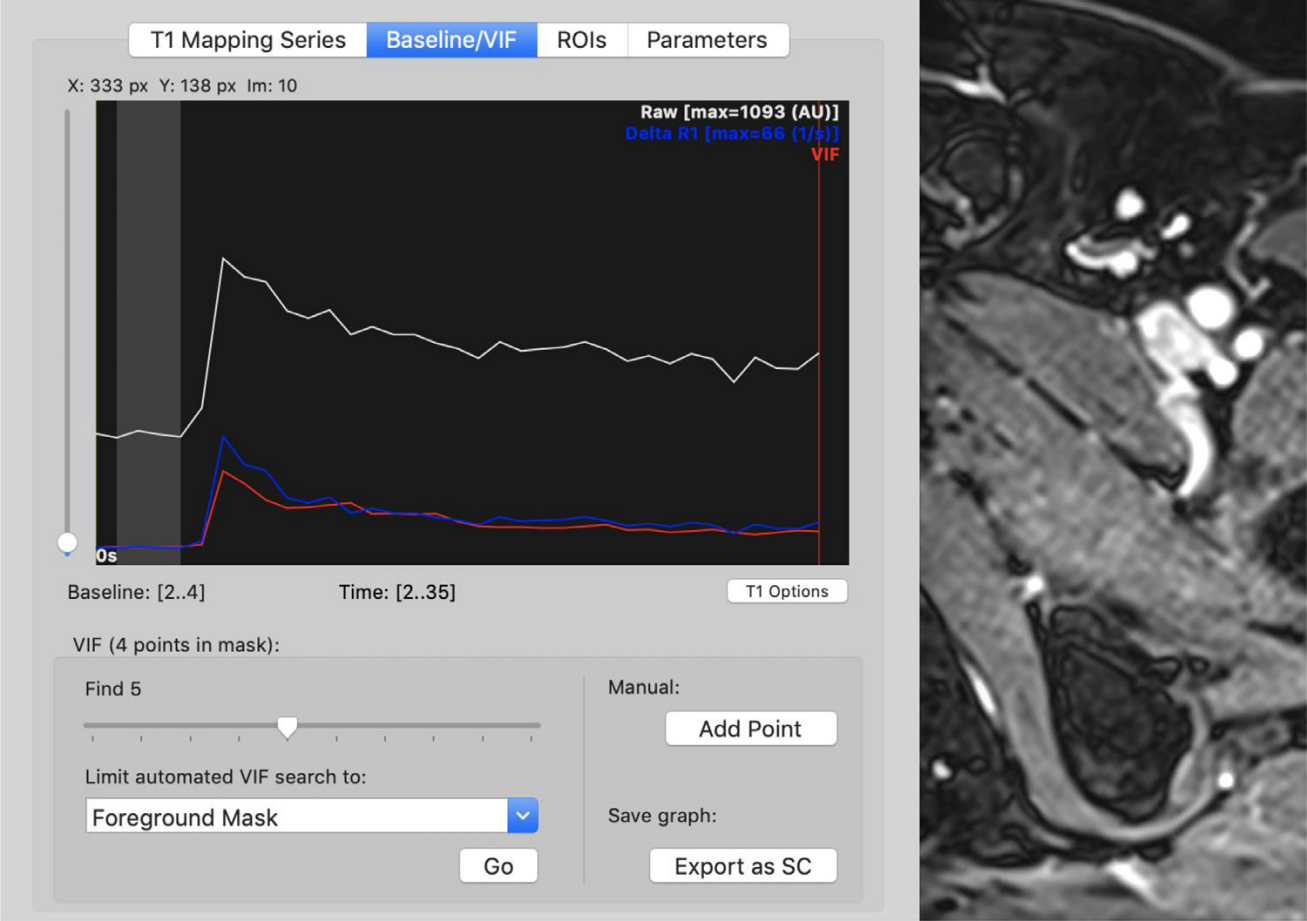
Manually selected ROI over the left femoral artery gave the VIF.

**Fig. 4:**
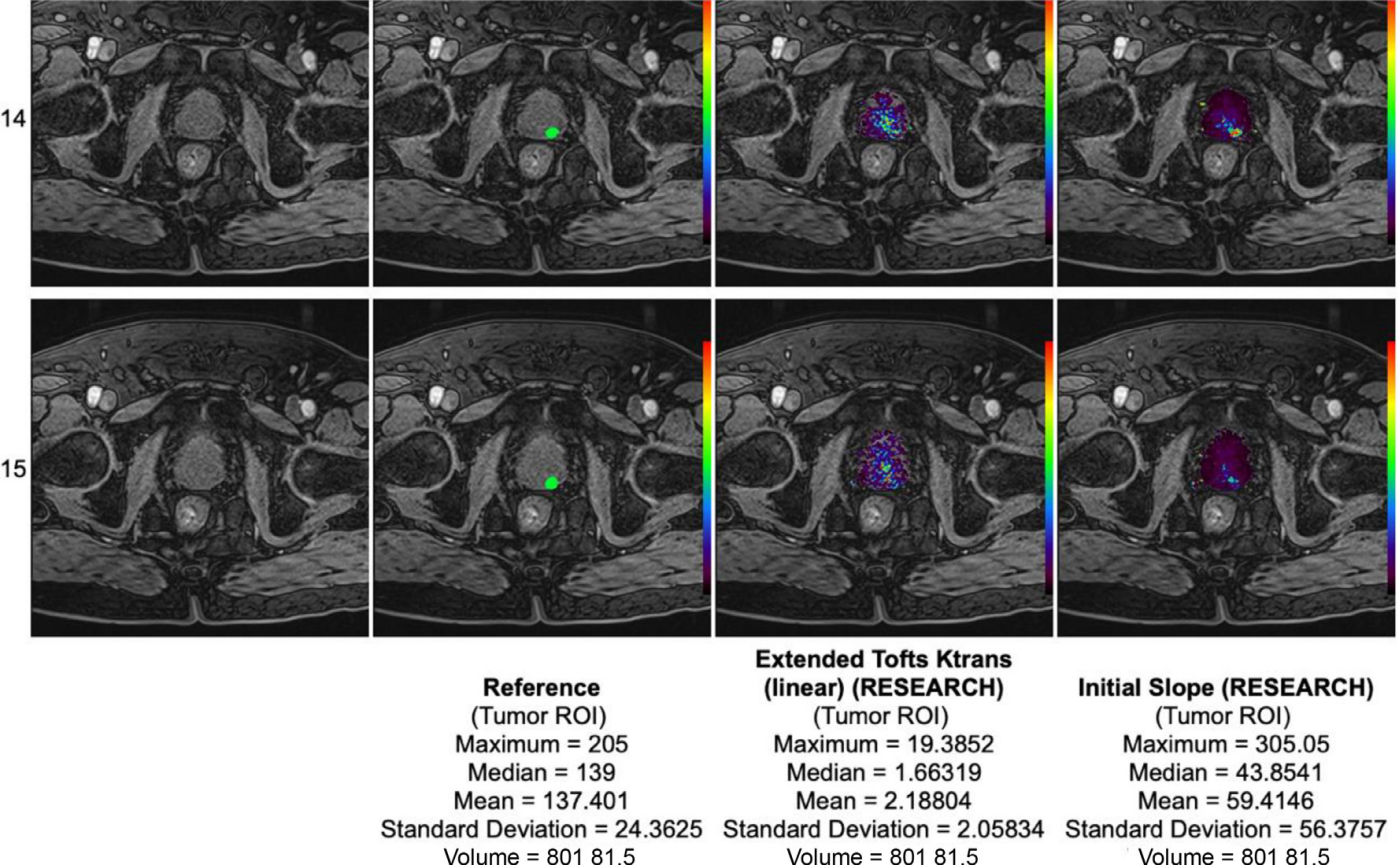
A PDF file with quantitative parameters and their values was generated for each case.

**Fig. 5:**
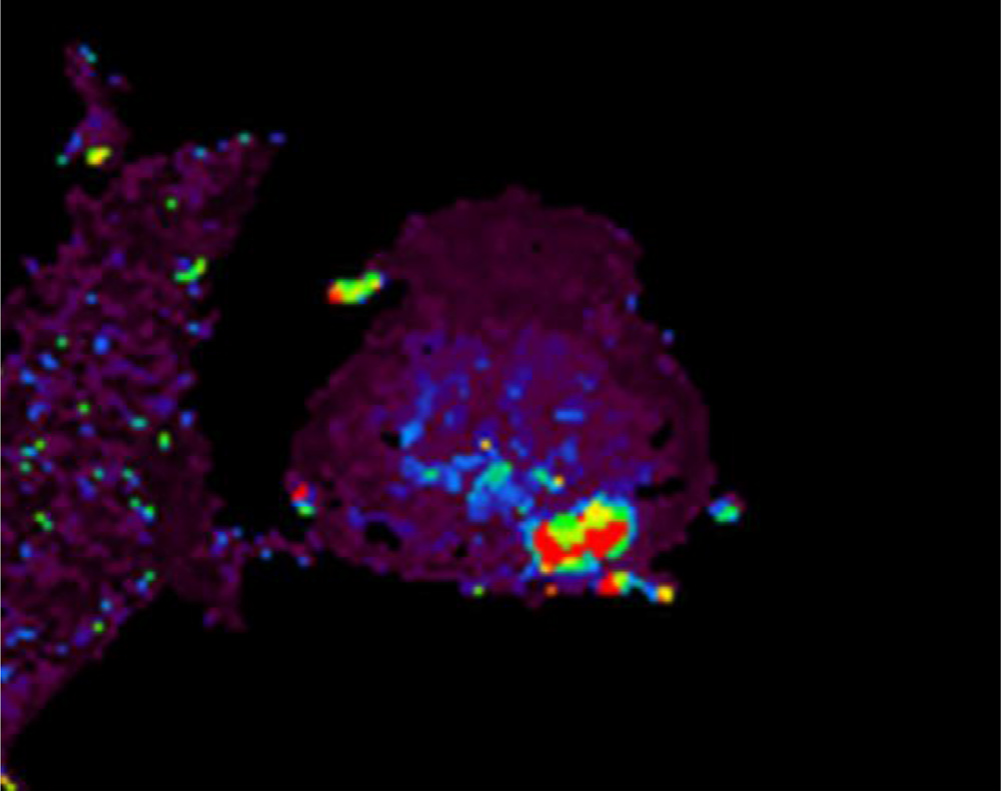
a map generated using the Initial Slope variable.

The following quantitative parameters were calculated using the IB DCE plugin for Horos DICOM Viewer (v2.0): Ktrans, Kep, Ve, Vp, Initial slope, Time to Peak, relative Time to Peak. Multiple series of the DCE images for each scan were opened in a 4D series. An ROI point was manually placed over the left femoral artery at the time when contrast entered the vessel, creating the vascular input function (VIF) (Fig. 3). To ensure the maps were calculated accurately, the ‘Length of Baseline’ represented by the vertical red line in the signal vs. time curve must be at the start of the upslope or ‘wash-in’ phase. The software used the initial ROI in order for the plugin to calculate the quantitative parameters (Fig. 4). The images of each model map were saved in the Horos DICOM system (Fig. 5).

### 2.4 Statistics

The primary outcome addresses the value of quantitative mpMRI parameters in addition to clinical factors in predicting focal salvage HIFU failure. ADC, Ktrans, Kep, Ve, Vp, Initial Slope, Time to Peak and relative Time to Peak were the quantitative parameters tested with the software. Composite recurrence free survival for the entire group was calculated using the Kaplan-Meier method. Univariable and multivariable Cox-regression analyses were performed for clinical and quantitative mpMRI data associated with time to focal salvage HIFU failure. Multiple imputation using the mice algorithm was performed for the missing mpMRI cases, assumed to be missing at random [13]. R version 3.5.3 was used for all statistical analyses. (R Foundation for Statistical Computing, Vienna, Austria;). All statistical tests had significance set at a p-value of < 0.05.

## 3 Results

A total of 89/150 (60%) men had pre-salvage HIFU mpMRI images available and were included in the study (Table 2). Prior to the ERBT the median PSA was 14 ng/ml (IQR 8.6-26.7). Low, intermediate and high-risk disease according to D'Amico criteria was documented in 21%, 26% and 42% respectively (11% missing data). The median follow-up was 35 months (IQR 23-47). Patient characteristics after ERBT and pre HIFU are shown in Table 2.

**Table 2:** Patients characteristics

Variable	*n*
*n* (%)	89
Age, median (IQR)	71 (65-74.5)
Time (months) to recurrence from primary treatment, median (IQR)	83 (64-110)
PSA pre-HIFU, median (IQR)	5.8 (3.8-8)
PSA DT pre-HIFU in months, median (IQR)	11.8 (7.5-17.5)
MRI tumor stage (%)	
T2	66 (74)
T3	23 (36)
Prostate volume, median, IQR)	26 (19-33.75)
Biopsy type pre-HIFU *n* (%)	
TPM	69 (78)
TRUS	20 (22)
ISUP grade pre-HIFU, *n* (%)	
1	3 (3)
2	46 (52)
3	25 (28)
4	9 (10)
5	6 (7)
D'Amico risk group pre-HIFU, *n* (%)	
Low	1 (1)
Medium	36 (40)
High	38 (43)
Tumour location pre-HIFU mpMRI, *n* (%)	
Posterior	71 (80)
Anterior	13 (15)
Both	2 (29
Type of HIFU, *n* (%)	
Focal ablation	62 (70)
Hemi-ablation	27 (30)
Neo-adjuvant ADT pre-HIFU *n* (%)	
No	50 (56)
Yes	39 (44)

39 patients received ADT treatment prior to salvage HIFU, prior to the intervention. ADT treatment was discontinued on the day of HIFU in all instances.

**Fig. 6:**
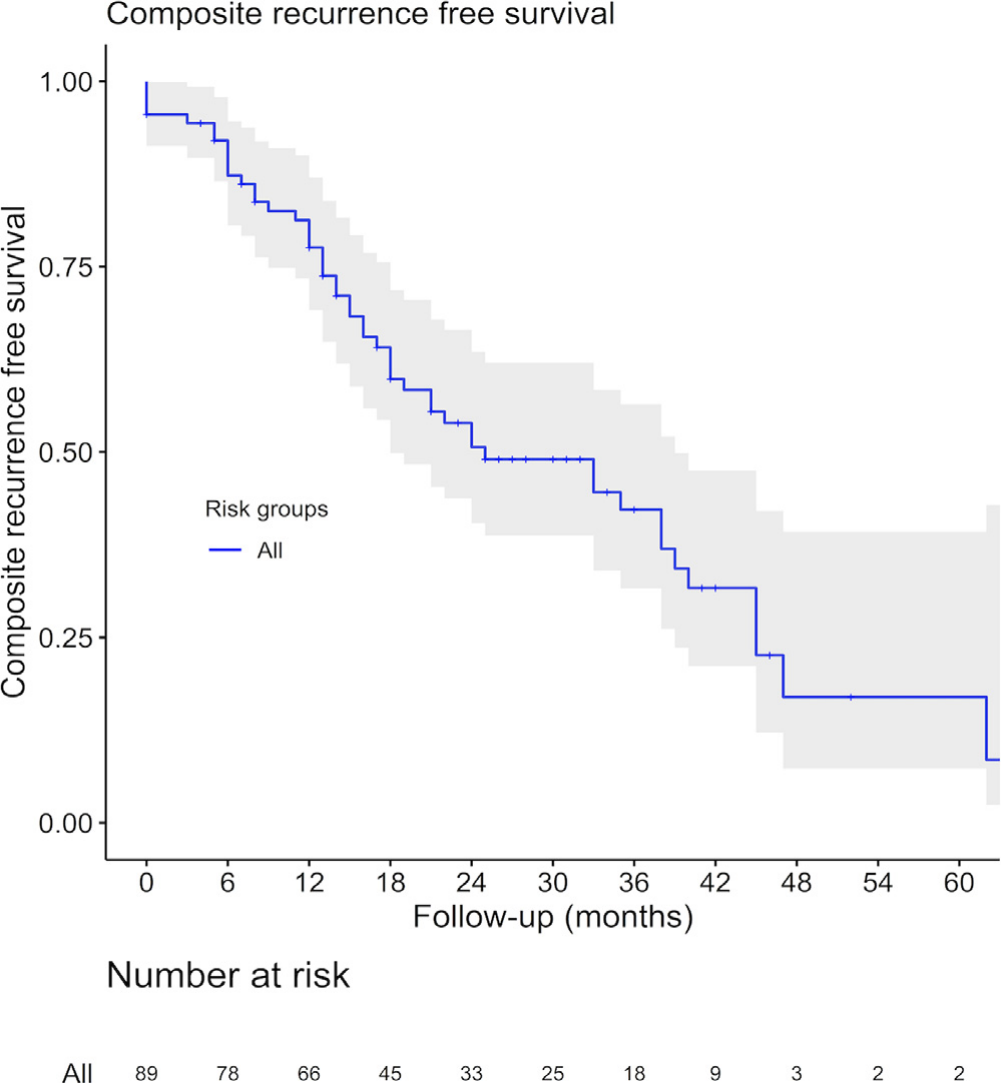
Kaplan Meier survival curve for composite outcome of the study population.

Overall there were 50/89 (56%) patients with recurrences with a total of 45/89 (51%) biochemical failures, 4/89 (4%) imaging failures and 1/89 (1%) positive biopsy. The median time to failure was 15 months (IQR 7.8 – 24.3). A Kaplan Meyer survival curve of the population is shown in Fig. 6. In the biochemical recurrence group (*n* = 45), the recurrence was confirmed in 7/45 (16%) patients with a biopsy, 13/45 (29%) had a positive bone scan and 11/45 (24%) suspicion of metastasis on a CT scan. 2/89 (2%) patients died during follow-up. There were 39/89 (44%) patients in the focal salvage HIFU success group.

One, 2 and 3 years composite recurrence free survival was 78% (95%-CI 69-87%), 51% (40-64%) and 42% (32-56%)

The basic mpMRI data like the tumor volume or the ADC value were available in all patients. The quantitative parameter analysis was performed in 49 (55%) patients. 40 (45%) patients were excluded due to mpMRI sequence incompatibility with IB DCE software.

At univariable analysis, from the quantitative mpMRI parameters, only the interstitial space volume, Ve mean and median values (HR 1.03, 95%CI 1.0-1.1, *P* = 0.032 and HR 1.0, 95%CI 1.0-1.1, *P* = 0.04) were predictors for composite outcome (Table 3). Clinical and MRI parameters that were predictive were PSA (HR 1.1, 95%CI 1.0-1.1, *P* = 0.01) and mpMRI prostate volume (HR 1.0, 95%CI 1.0-1.03, *P* = 0.04). In the multivariable analysis, only Ve median value (HR 1.04 95%CI 1.0-1.1, *P* = 0.01) remained as an independent predictor for progression (Table 4).

**Table 3:** Univariate analysis of potential predictors for composite outcome

Univariable analysis				
Variable	HR	Lower 95%-CI	Upper 95%-CI	*P* value
MRI prostate volume	1.02	1.001	1.03	0.04
T-stage 3 vs. 1+2	1.4	0.8	2.5	0.3
Grade group 3 (vs. 1+2)	1.0	0.5	1.9	0.9
Grade group 4 (vs. 1+2)	0.6	0.2	2.1	0.4
Grade group 5 (vs. 1+2)	2.1	0.7	6.1	0.2
PSA	1.1	1.0	1.1	0.01
PSADT	1.0	0.9	1.0	0.1
Biopsy type (TPM vs. TRUS)	1.5	0.8	2.7	0.2
ROI volume	1.3	0.8	2.1	0.3
ADC	1.0	1.0	1.0	0.4
KTrans median	1.1	0.8	1.5	0.6
KTrans mean	1.1	0.8	1.3	0.6
Kep median	0.9	0.8	1.0	0.2
Kep mean	0.9	0.8	1.1	0.3
Ve median	1.03	1.003	1.06	0.03
Ve mean	1.03	1.002	1.06	0.04
Vp median	1.0	0.9	1.2	0.5
Vp mean	1.0	0.9	1.1	0.7
IS median	0.9	1.0	1.0	0.4
IS mean	0.9	1.0	1.0	0.1
rTTP median	1.0	1.0	1.0	0.3
rTTP mean	1.0	1.0	1.0	0.3
TTP median	1.0	1.0	1.0	0.3
TTP mean	1.0	1.0	1.0	0.3

**Table 4:** Multivariable analysis of potential predictors for composite outcome

Multivariable analysis				
Variable	HR	Lower 95%-CI	Upper 95%-CI	*P* value
Ve Median	1.04	1.01	1.07	0.01
T-stage 3 vs. 1+2	1.4	0.7	2.7	0.3
Grade group 3 (vs. 1+2)	0.9	0.4	1.8	0.7
Grade group 4 (vs. 1+ 2)	0.4	0.1	1.4	0.2
Grade group 5 (vs. 1+2)	2.4	0.8	7.6	0.1
PSA	1.1	1.0	1.1	0.1
ROI volume	1.0	0.6	1.7	1.0
MRI prostate volume	1.0	1.0	1.0	0.06

## 4 Discussion

Our study evaluated whether mpMRI pharmacokinetic quantitative parameters can predict failure in patients undergoing salvage HIFU treatment for radioreccurent PCa. Our results show that the extracellular space of the tumor, represented by Ve value, measured in the DCE sequence of the mpMRI, is an independent predictor of failure, corrected for well-known clinical parameters associated with treatment failure. Interestingly, in our cohort, tumor grade was not a predictor and it may indicate, that the critical factor is the tumor micro-environment in the success or otherwise of salvage HIFU treatment.

Numerous studies have analyzed the correlation of pharmacokinetic parameters with the aggressiveness of the cancer in untreated disease. For example, Wei et al. prospectively included 150 men with suspected PCa and found that quantitative parameters showed a significant difference between benign and cancerous lesions [14]. The mean Ktrans value could also distinguish between low-grade and high-grade cancer. Results by Ocak et al. also confirm these findings with higher Ktrans and Kep values in suspicious lesions compared to non-suspicious peripheral zone. In radiorecurrent cancer interesting data is emerging with quantitative parameters and PCa. Fernandes et al. analyzed the ability of quantitative parameters to delineate the tumor extent in mpMRI in comparison to the final pathology, and showed that T2-weighted, ADC, Ktrans and kep had the best performance in distinguishing tumor and benign voxels [10]. In our findings, Ktrans and Kep did not correlate significantly with the outcomes of the patients, with Ve value being the most significant factor for failure in the follow-up.

Gleason score was not a predictor for recurrent disease in our cohort. In a study by Dason et al. which analyzed predictors for salvage HIFU failure with a similar composite endpoint, Gleason score was also not a significant predictor for treatment failure [15]. In contrast, another study including 290 after salvage HIFU, concluded that Gleason ≥8 vs. ≤6 (HR: 1.17, 95%CI 1.03-1.3), was a significant predictor for progression free survival. However, the interpretation of histology after ERBT can be challenging [16]. Radiotherapy causes cytologic atypia of benign glands, forcing the pathologist to discriminate cancer mostly on architectural findings. These changes vary widely among patients and might influence the grading. Up to 35% of Gleason scores in post radiotherapy patients can be underestimated and 14% overestimated [17]. There was also no difference concerning the biopsy approach (TRUS vs. TPM) and the outcome. Our hypothesis is that in most cases prostate size was significantly reduced after ERBT resulting in more precision when performing TRUS biopsies.

Our literature review did not yield any studies that evaluated mpMRI quantitative parameters for predicting outcomes of salvage HIFU for treating radiorecurrent PCa. However, studies done in other solid organ cancers show such quantitative parameters might improve pre-operative risk stratification. Fasmer et al. compared the value of quantitative parameters with the outcomes in endometrial cancer patients [18]. In their findings, Kep was associated with high-risk histological subtype (*P* = 0.04 and poor prognosis (*P* = 0.09). Similar findings are also reported in studies analyzing breast, colorectal, liver, head and neck cancers and osteosarcomas [19].

The main limitation of the presented analysis is the sample size where by 150 patients were reduced to 89 mostly for inadequate scans for the reasons detailed in the exclusion criteria and of those 55% were analyzable for quantitative parameters. Some of the mpMRI scans were external films and there was inherent variability in the scan quality. Although, mpMRI protocols improved over the study period due to our involvement in trials such as PROMIS, these were performed to a high standard throughout the study period. In addition, for our presented analysis only MRI scans of adequate quality were included. The resulting small sample size meant the study was underpowered and may not have represented the true effect of a number of other factors. Also, a single center design could influence the accuracy of our results. Our findings require external validation. Increasing the number of patients will inevitably increase the power of the study and will allow a multivariate model to be tested and establish whether the findings drawn here are replicable. Extending this research model into other modalities of focal therapy such as cryotherapy, irreversible electroporation and photodynamic therapy, as well as within the primary setting for PCa will be necessary. Finally, a proportion of patients had ADT prior to their HIFU treatment, which could have been a confounder when analyzing biochemical failure as the actual pre-HIFU PSA levels would have been artificially low. This occurred as many referring oncologists placed patients on ADT prior to referral for salvage therapy. We took the PSA prior to ADT. It is unlikely that ADT had a significant impact for our results, since majority of the patients received the ADT for a short duration after confirmation of recurrence and the HIFU treatment.

## 5 Conclusion

One pharmacokinetic quantitative parameter based on DCE sequences seems to independently predict failure following focal salvage HIFU for radiorecurrent prostate cancer. This likely relates to the tumor microenvironment producing heat-sinks which counter the heating effect of HIFU. Further validation in larger datasets and evaluating mechanisms to reduce heat-sinks are required.

## Funding sources

Wellcome Trust, funding of open access publication

## Conflicts of Interest

None
